# Impact of Beer and Nonalcoholic Beer Consumption on the Gut Microbiota: A Randomized, Double-Blind, Controlled Trial

**DOI:** 10.1021/acs.jafc.2c00587

**Published:** 2022-06-15

**Authors:** Cláudia Marques, Liliana Dinis, Inês Barreiros Mota, Juliana Morais, Shámila Ismael, José B. Pereira-Leal, Joana Cardoso, Pedro Ribeiro, Helena Beato, Mafalda Resende, Christophe Espírito Santo, Ana Paula Cortez, André Rosário, Diogo Pestana, Diana Teixeira, Ana Faria, Conceição Calhau

**Affiliations:** † Nutrição e Metabolismo, Faculdade de Ciências Médicas/NOVA Medical School, Universidade NOVA de Lisboa, Lisboa 1169-056, Portugal; ‡ CINTESIS-Center for Health Technology Services Research, Faculdade de Ciências Médicas/NOVA Medical School, Universidade NOVA de Lisboa, Lisboa 1169-056, Portugal; § CHRC-Comprehensive Health Research Centre, CEDOC-Chronic Diseases Research Center, Faculdade de Ciências Médicas/NOVA Medical School, Universidade NOVA de Lisboa, Lisboa 1169-056, Portugal; ∥ Ophiomics—Precision Medicine, Lisboa 1600-514, Portugal; ⊥ Centro de Medicina Laboratorial Germano de Sousa, Lisboa 1600-513, Portugal; # CATAA—Centro de Apoio Tecnológico Agro Alimentar, Castelo Branco 6000-459, Portugal; ∇ Unidade Universitária Lifestyle Medicine José de Mello Saúde by NOVA Medical School, Lisboa 1169-056, Portugal

**Keywords:** alcohol, beer, gut microbiota, nonalcoholic beer, polyphenols

## Abstract

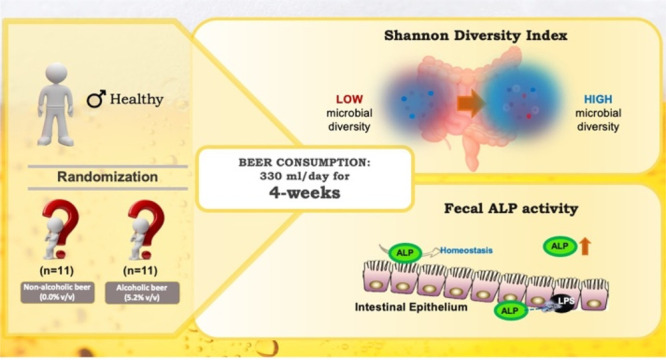

Gut microbiota modulation might constitute a mechanism mediating the effects of beer on health. In this randomized, double-blinded, two-arm parallel trial, 22 healthy men were recruited to drink 330 mL of nonalcoholic beer (0.0% v/v) or alcoholic beer (5.2% v/v) daily during a 4-week follow-up period. Blood and faecal samples were collected before and after the intervention period. Gut microbiota was analyzed by 16S rRNA gene sequencing. Drinking nonalcoholic or alcoholic beer daily for 4 weeks did not increase body weight and body fat mass and did not changed significantly serum cardiometabolic biomarkers. Nonalcoholic and alcoholic beer increased gut microbiota diversity which has been associated with positive health outcomes and tended to increase faecal alkaline phosphatase activity, a marker of intestinal barrier function. These results suggest the effects of beer on gut microbiota modulation are independent of alcohol and may be mediated by beer polyphenols.

## Introduction

Beer, a fermented extract of malted barley grains, is the most widely consumed alcoholic beverage in the world. The consumption of low to moderate doses of beer is protective against cardiovascular risk, as shown by epidemiologic studies, and such protective effects are comparable to that reported for moderate wine consumption.^1^

The ethanol content of a beer generally varies from 3.5 to 10% w/v.^1^ According to the 2020–2025 Dietary Guidelines for Americans, if alcohol is consumed, it should be in moderation—up to one drink per day (14 g of alcohol) for women and up to two drinks per day (28 g of alcohol) for men—which typically comprises one or two bottles of beer (330 mL) with 4% w/v alcohol. Numerous mechanisms have been proposed to mediate the protective effects of some fermented alcoholic beverages in cardiovascular disease including an increase in high-density lipoprotein (HDL) cholesterol, a decrease in low-density lipoprotein (LDL) cholesterol, a reduction in platelet aggregation, and an increase in insulin sensitivity.^2^ Nevertheless, the protective effects of alcoholic beverages for ischemic heart disease and diabetes are offset by the association of alcohol consumption with cancer.^3^ Thus, despite the number of molecular and preclinical studies showing the benefits of fermented alcoholic beverages it is important to investigate and compare the effects of alcoholic and dealcoholized beer (beer from which the ethanol content has been removed after fermentation).

Beer with an “alcoholic strength by volume” (ABV) not exceeding 0.5% is considered nonalcoholic or alcoholic free beer, in some parts of the European Union, but in the U.K., for instance, alcohol-free beer can contain no more than 0.05% ABV.

Besides the alcoholic content, beer is the main (and probably the only) source of hop polyphenols in the human diet. Hops are almost exclusively used by the beer production industry to confer beer aroma and bitterness, but they also contain interesting amounts of prenylflavonoids, namely xanthohumol.^4^ Several preclinical studies suggest that xanthohumol lowers the risk of the development and progression of oxidative stress-related diseases, such as chronic diseases, including obesity and diabetes.^5^ During the brewing process, xanthohumol undergoes a ring-closing reaction, being converted (isomerized) into isoxanthohumol which also has biological activity.^4^

Similar to other classes of phenolic compounds, beer polyphenols might reach the gut where they can modulate bacterial growth. In addition, some beers may contain live fermentation microorganisms. The Flemish Gut Flora Project, one of the largest population-wide studies to assess the variation of gut microbiota among healthy individuals, has shown that beer consumption is a key influence on the overall microbiota composition.^6^ Therefore, given the importance of the gut microbiota in the pathophysiology of obesity, cardiovascular disease, and diabetes, gut microbiota modulation might constitute another mechanism mediating the effects of beer on health.^7^

The lack of randomized clinical trials studying the effect of moderate alcoholic and nonalcoholic beer consumption on intermediate markers of cardiovascular risk and on gut microbiota encouraged the present study.

The aim of this pilot study was to evaluate the effect of beer with alcohol (5.2%) and without alcohol (0.0%) on cardiometabolic markers and gut microbiota composition in healthy men.

## Materials and Methods

### Participants

Healthy volunteers were recruited from the Lisbon metropolitan area through social media advertising. Volunteers were invited to visit NOVA Medical School for a physical examination and a brief questionnaire about their medical history in order to determine their eligibility to participate in the study.

Inclusion criteria included healthy men, moderate alcohol consumers, aged 18 to 65 years old, free of chronic diseases with relevant effect on gastrointestinal system (including functional bowel disorders), willing and able to provide written informed consent. Exclusion criteria were as follows: documented cardiovascular disease (ischemic heart disease—angina or recent or old myocardial infarction or previous or cerebral vascular incident, peripheral vascular disease); diabetes or other relevant metabolic diseases; infectious diseases, namely infections with HIV, Hepatitis B or C virus; intake of antibiotics in the last 4 weeks or laxatives in the last 2 weeks; and subjects with history of drug, alcohol, or other substances abuse.

All participants signed their written informed consent after receiving oral and written information about the study. This trial was approved by the Ethics Committee of NOVA Medical School. This trial is registered at clinicaltrials.gov as NCT03513432.

### Study Design and Protocol

To investigate the effect of beer alcohol content on markers of cardiometabolic risk and gut microbiota composition, a 4-week randomized, double-blinded, two arm parallel-group pilot trial was conducted at NOVA Medical School.

Participants were randomly assigned into one of the intervention groups (ratio 1:1) using a computer-generated allocation sequence. The intervention comprised the daily consumption of 330 mL beer with 0.0% alcohol (Group A) and 330 mL beer with 5.2% alcohol (Group B).

Given the stability and resilience of the human gut microbiota, a two-arm parallel design was chosen to minimize the risk of a carryover effect between interventions.

The participants and the research team were blinded to the study beers. Beers were delivered to each participant without the original label, with a label code (A/B). Only one researcher (from another institution) knew the correspondence code. At the end of the study, participants were asked about which beer they thought they had consumed. The unblinding was performed after statistical analysis was completed.

### Intervention

Participants were instructed to not change their physical activity levels and maintain their dietary habits (including alcohol consumption) throughout the study.

After a run-in period of 1 week, at baseline, participants visited the research center for blood collection, body composition evaluation, and faecal samples delivery. Volunteers were instructed to arrive after a 12 h overnight fast. Body composition was evaluated using simultaneous multifrequency bioelectrical impedance analysis (InBody770, InBody Europe, Amsterdam, The Netherlands). Mediterranean diet adherence screener (MEDAS) was applied at baseline to characterize participants’ dietary pattern.^8^ Alcohol habits were evaluated using the appropriate questions from the semiquantitative food frequency questionnaire, previously validated for the Portuguese population.^9^ After 4 weeks, participants returned for a second visit which followed identical procedures. In the second visit, participants were asked individually to honestly report if they had changed their dietary habits or physical activity levels during the study.

Beer was provided weekly in a package containing 7 beer bottles. Participants were advised to drink the study beer daily at dinner and to take the necessary precautions since it could contain alcohol (the study was double-blind). Compliance to the study protocol (daily consumption of beer) was monitored weekly through self-reported questionnaires. Participants were asked to honestly report whether they forgot to drink the study beer on one or more days of the week, each week. If participants drank the study beer every day during the 4 weeks, compliance was considered 100%.

### Beer

Lager beer was provided by Super Bock Group (Leça do Balio—Matosinhos, Portugal). The nutritional composition of the beer was analyzed by Silliker Portugal, S.A. Xanthohumol and isoxanthohumol were measured by HPLC-DAD after SPE extraction, in the Faculty of Pharmacy of University of Porto, and the total phenolics were determined by the Folin-Ciocalteu method.^10^

### Outcomes

The primary outcome of the study were the changes in intestinal microbiota from baseline and the secondary outcomes were the changes in body mass index, total body fat mass, homeostasis model assessment-insulin resistance (HOMA-IR), fasting serum total cholesterol, fasting serum HDL cholesterol, fasting serum LDL cholesterol, and fasting serum triglycerides.

### Biochemical Analysis

Venous blood samples were collected by venepuncture into serum separator tubes (BD Vacutainer SST II Advance, Becton Dickinson). Glucose, insulin, total cholesterol, HDL cholesterol, LDL cholesterol, aspartate aminotransferase (ASAT) activity, alanine aminotransferase (ALAT) activity, alkaline phosphatase (ALP) activity, gamma-glutamyl transferase (GGT) activity, C-reactive protein (CRP), homocysteine, sodium, and potassium were measured in serum. HOMA-IR was calculated using the following formula: insulin (mU/L) * glucose (mg/dL)/405. For measuring glycated hemoglobin (HbA1c), blood was collected into tubes containing K_2_-EDTA (BD Vacutainer, Becton Dickinson). Biochemical evaluation of samples was performed in an outsourced certified medical laboratory (BMAC—Análises Clínicas and Centro de Medicina Laboratorial Germano de Sousa).

### Gut Microbiota Characterization

Fecal samples were collected by volunteers at some time point up to 48 h before each visit. Samples were collected with the stool collection kit provided (EasySampler, ALPCO) containing RNAlater (Sigma-Aldrich). Samples were kept at −20 °C until DNA extraction. Genomic DNA was extracted and purified from stool samples using NZY Tissue gDNA Isolation Kit (NZYTech) as previously described by Marques et al.^11^

All 16S DNA libraries (V3 and V4 regions) were prepared, sequenced, and analyzed in accordance with the manufacturer’s instructions, for each kit and instrument, as previously described in Moreira-Rosário et al.^12^

### Fecal Alkaline Phosphatase (ALP) Activity

ALP activity was determined in fecal samples as a marker of intestinal inflammation and permeability,^13^ as previously described by Ismael et al.^14^

### Statistical Analysis

Statistical analysis was performed using SPSS V.23 software. Data are expressed as mean ± standard deviation. Differences were considered statistically significant when *P* < 0.05. A Mann-Whitney test was used to compare baseline characteristics of study participants in each group. A Wilcoxon signed-rank test was used to compare the differences between baseline and postintervention period (within groups). Changes during 4-week intervention period, between groups, were compared by Mann-Whitney test. Correlation between variables was established using Spearman’s correlation test.

The microbiome data analysis was performed using Microbiome Analyst—a web-based tool for comprehensive statistical, visual, and meta-analysis of microbiome data.^15^ Alpha diversity was measured by Shannon’s diversity index that summarizes both the species richness (total number of species) and evenness (abundance distribution across species) within a sample. To evaluate beta-diversity, a principal coordinates analysis (PCoA) plot based upon Bray–Curtis dissimilarity was created to evaluate differences in the community of bacterial genus according to the experimental factor–Timing (Baseline and Final), in each group. The distances (or dissimilarity) between samples of the same group were compared to the distances between groups using PERMANOVA.

The number of subjects in a group was chosen based on other works evaluating the impact of fermented beverages on the gut microbiota (Queipo Ortuño, 2012). Sample size calculated a posteriori shows that our study with about 10 participants per group, provide 80% power (alpha = 0.05) to detect a change of 14% between groups in Shannon’s diversity index.

## Results

### Participants

**Figure 1:**
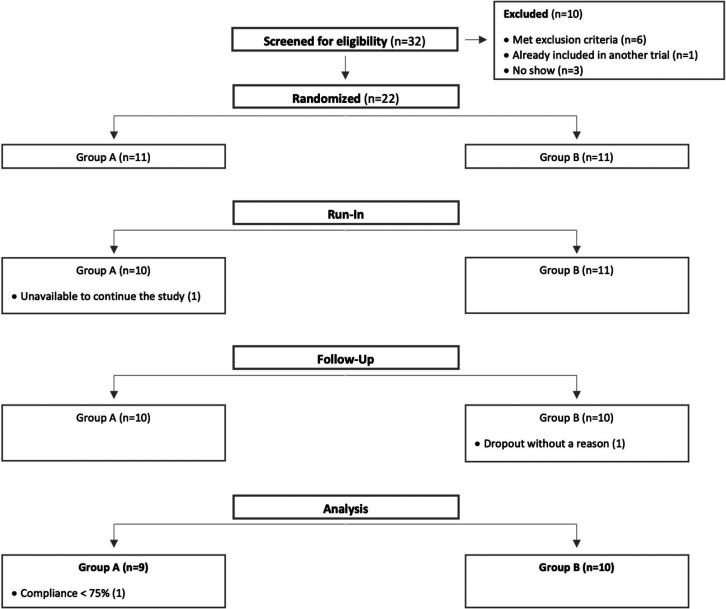
Trial flowchart.

From the 32 subjects screened for eligibility to participate in this pilot study, 22 were randomly assigned to one of the intervention groups (*n* = 11 in each group). During the study, two individuals dropped out: one was unavailable to continue the study due to personal reasons and one did not show up in the follow-up visits for unspecified reasons (Figure 1). A modification to the initial trial protocol was made since the number of participants recruited was not enough for having an identical third arm where the impact of nonalcoholic beer with 0.5% v/v would be evaluated.

Study participants were healthy men, with a mean age of 35 years (range: 23–58 years). At baseline, the usual alcohol consumption of participants from group A and B was 11.1 ± 6.8 and 11.30 ± 13 g/day (*P* = 0.606), respectively. Participants from group A and B had a moderate adherence to the Mediterranean Diet as shown by the MEDAS score of 8 ± 2 and 8 ± 2 (*P* = 0.720), respectively. Participants did not report any significant change in their dietary habits during the study. Baseline characteristics of study participants in each group, including metabolic markers and gut microbiota composition, did not differ significantly at baseline (*P* > 0.05).

### Beer

The nutritional composition of the beers under study are available in Table S1 of the Supporting Information (SI). The three beers under study had a similar phenolic composition (Table S1). Xanthohumol was not detected in beer since, during the brewing process, xanthohumol is isomerized into isoxanthohumol (Table S1). The only main difference found between the study beers was the alcoholic content.

### Compliance and Blinding

Weekly questionnaires revealed good compliance to the study protocol except for one volunteer. This participant was excluded from the per protocol analysis since compliance was below 75% (Figure 1). Although the study was double-blind, 80% of participants in group B reported they were drinking alcoholic beer, whereas 89% of participants in group A reported they were drinking nonalcoholic beer.

### Metabolic Markers

Drinking nonalcoholic beer or alcoholic beer daily for 4 weeks did not increase body weight and body fat mass. It also did not change cardiometabolic biomarkers such as glucose, Hb A1C, insulin, HOMA-IR, cholesterol (LDL and HDL), serum triglycerides, CRP, and homocysteine (Table 1). Nevertheless, nonalcoholic beer increased total cholesterol, although the levels remain below 200 mg/dL, apparently due to the high variation observed in this group for serum triglycerides levels.

**Table 1:** Effect of 4-Week Beer Intake (with and without Alcohol) on Hepatic, Inflammatory, and Metabolic Markers^a^^,^^b^

	beer 0.0% alcohol (*n* = 9) Group A			beer 5.2% alcohol (*n* = 10) Group B			
	baseline	post-intervention	*P*^c^ within group	baseline	post-intervention	*P*^c^ within group	*P*^d^^2^ variation between groups
BMI (kg/m^2^)	26.3 ± 6.7	26.1 ± 6.7	0.088	25.2 ± 3.7	25.2 ± 3.9	0.833	0.182
BFM (kg)	21.5 ± 17.4	21.6 ± 17.1	0.722	14.3 ± 8.8	13.9 ± 8.8	0.090	0.113
glucose (mg/dL)	80.0 ± 9.0	78.7 ± 6.1	0.673	83.5 ± 10.2	81.0 ± 10.1	0.237	0.661
Hb A1C (%)	5.1 ± 0.3	5.1 ± 0.2	1.000	5.4 ± 0.4	5.2 ± 0.3	0.172	0.182
insulin (μU/mL)	7.6 ± 4.0	9.1 ± 9.1	0.594	5.4 ± 2.3	5.8 ± 3.9	0.759	0.762
HOMA-IR	1.5 ± 0.9	1.6 ± 2.0	0.594	1.1 ± 0.5	1.2 ± 0.8	0.721	0.549
sodium(mmol/L)	142.3 ± 2.1	141.2 ± 1.4	0.079	142.3 ± 1.3	141.3 ± 1.1	0.047*	0.661
potassium(mmol/L)	4.4 ± 0.3	4.5 ± 0.4	0.172	4.3 ± 0.3	4.8 ± 0.4	0.009*	0.079
total chol (mg/dL)	179.9 ± 37.3	195.3 ± 37.1	0.038*	178.3 ± 36.5	178.2 ± 40.0	0.683	0.053
HDL (mg/dL)	49.1 ± 12.4	47.6 ± 12.8	0.212	50.3 ± 3.7	49.3 ± 3.6	0.766	0.780
LDL (mg/dL)	104.6 ± 28.7	114.7 ± 27.1	0.260	107.4 ± 28.4	112.0 ± 33.4	0.263	0.549
triglycerides (mg/dL)	132.1 ± 92.3	174.2 ± 188.8	0.441	103.2 ± 81.4	84.4 ± 55.0	0.074	0.156
homocysteine (μmol/L)	12.9 ± 4.2	12.8 ± 3.0	0.674	10.8 ± 3.4	12.3 ± 3.6	0.169	0.113
ASAT (U/L - 37°)	30.8 ± 5.8	30.9 ± 6.3	0.944	31.6 ± 6.7	54.9 ± 73.8	0.905	0.842
ALAT (U/L - 37°)	33.2 ± 12.5	33.4 ± 17.3	0.406	35.6 ± 15.9	46.8 ± 32.9	0.779	0.604
ALP (U/L - 37°)	74.6 ± 15.1	71.4 ± 13.1	0.028*	84.5 ± 22.7	78.6 ± 17.4	0.015*	0.356
GGT (U/L - 37°)	37.8 ± 23.3	47.6 ± 40.1	0.085	24.8 ± 12.3	28.0 ± 16.4	0.759	0.356
CRP (mg/dL)	0.4 ± 0.6	0.5 ± 0.9	0.249	0.1 ± 0.1	0.2 ± 0.2	1.000	0.156

a Data are expressed as mean ± standard deviation. *P < 0.05 vs respective baseline.

b BMI, body mass index; BFM, body fat mass; HbA1C, glycated hemoglobin; HOMA-IR, homeostasis model assessment for insulin resistance; Total Chol, total cholesterol; HDL, high-density lipoprotein; LDL, low-density lipoprotein; ASAT, aspartate aminotransferase; ALAT, alanine aminotransferase; ALP, alkaline phosphatase; GGT, gamma-glutamyl transferase; CRP, C-reactive protein.

c Statistical analysis was performed using Wilcoxon signed-rank test.

d Statistical analysis was performed using Man-Whitney test.

Interestingly, hepatic transaminases, such as ASAT, ALAT, and GGT, were not increased by daily consumption of alcoholic beer (Table 1). On the contrary, alkaline phosphatase (ALP), a marker of hepatic, kidney, or bone injury was decreased after 4 weeks of daily beer intake, independently of beer ethanol content (Table 1).

After 4-weeks, in the volunteers drinking alcoholic beer, serum potassium levels were increased, whereas sodium levels were decreased (Table 1). The same trend was observed for nonalcoholic beer. Nevertheless, these effects may not be clinically relevant since the sodium and potassium levels are still within the reference values for adults.

### Gut Microbiota Composition

**Figure 2:**
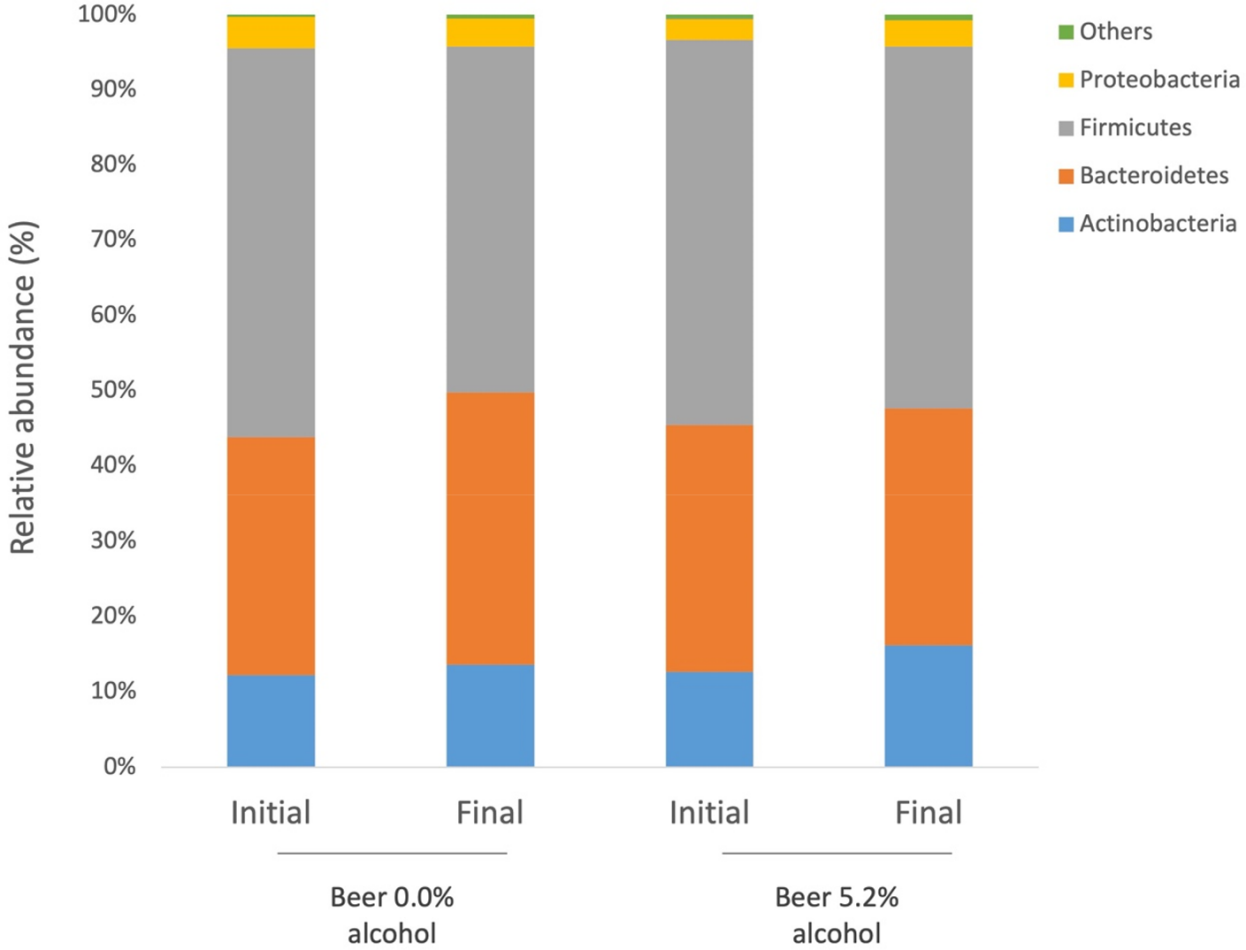
Gut microbiota composition at the phylum level in the two intervention groups, at baseline (initial) and 4 weeks after intervention (final). Bars represent the average of each bacterial phylum relative abundance. Each phylum is represented by a different color.

**Figure 3:**
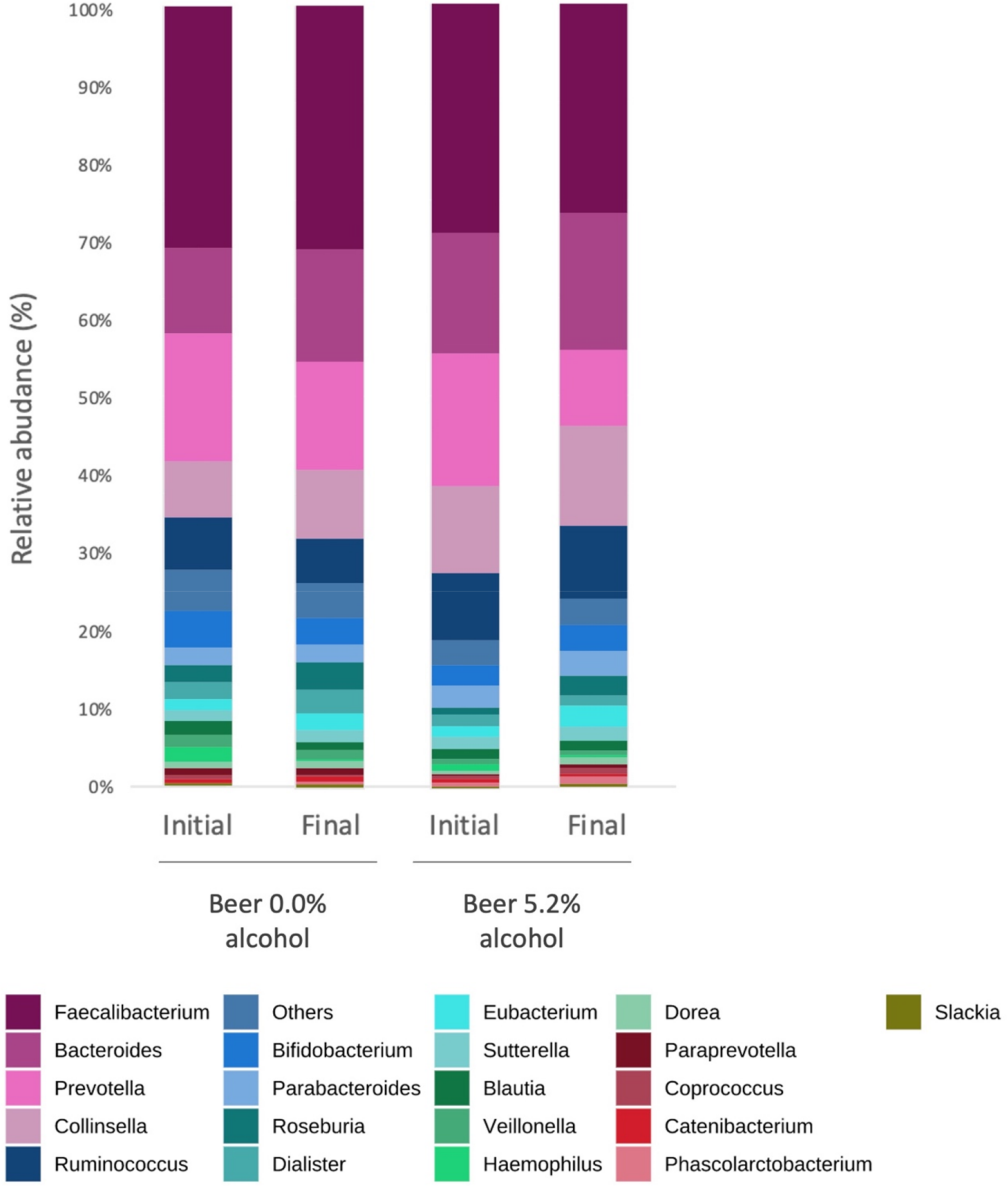
Gut microbiota composition at the genus level in the two intervention groups, at baseline (initial) and 4 weeks after intervention (final). Bars represent the average of each bacterial genus relative abundance. Each genus is represented by a different color.

Results illustrated in Figures 2 and 3 indicate, respectively, the phylum and genus level distribution of participants’ gut microbial communities, before and after intervention. Overall, the most abundant bacterial phyla at baseline in group A (nonalcoholic beer) and B (alcoholic beer) were Firmicutes (52% and 46%, respectively), followed by Bacteroidetes (32% and 36%, respectively), and Actinobacteria (12% and 14%, respectively). The most abundant bacterial genera at baseline in group A and B were Faecalibacterium (30% and 28%, respectively), followed by Prevotella (16% and 16%, respectively), Bacteroides (11% and 15%, respectively), and Collinsella (7% and 10%, respectively).

**Figure 4:**
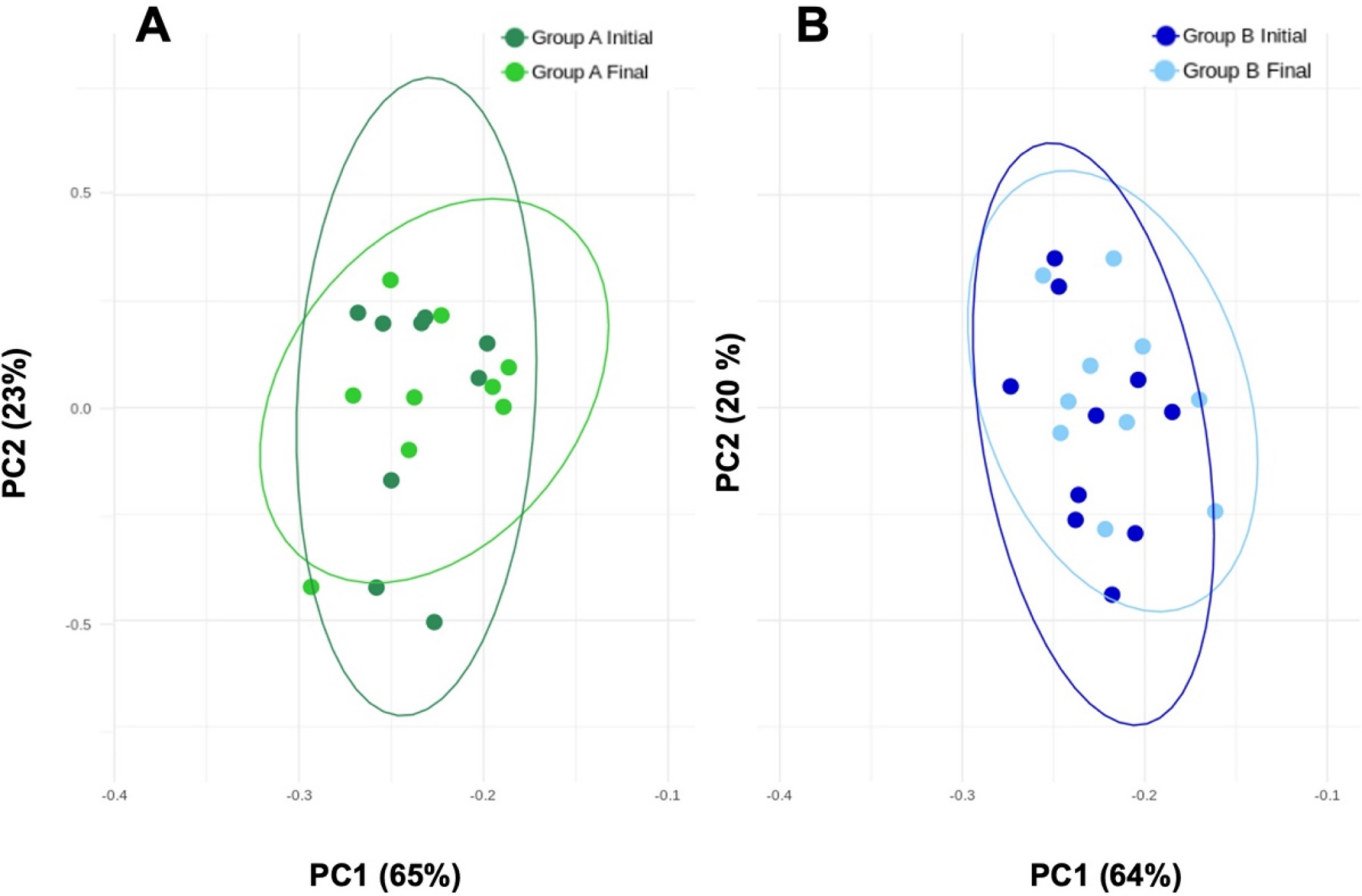
Gut bacterial genera were clustered using principal component analysis (PCA). Results are plotted according to the first two principal components, which explain 65% (PC1) and 23% (PC2), 64% (PC1) and 20% (PC2) of the variation of the gut microbiota composition during the intervention (A) beer 0.0% alcohol beer and (B) beer 5.2% alcohol, respectively. Each point represents one sample. Circles combine samples collected at the same time point by their respective 95% confidence interval ellipse. No differences (*p* > 0.05) were observed, suggesting similarity among the groups clustered together.

**Figure 5:**
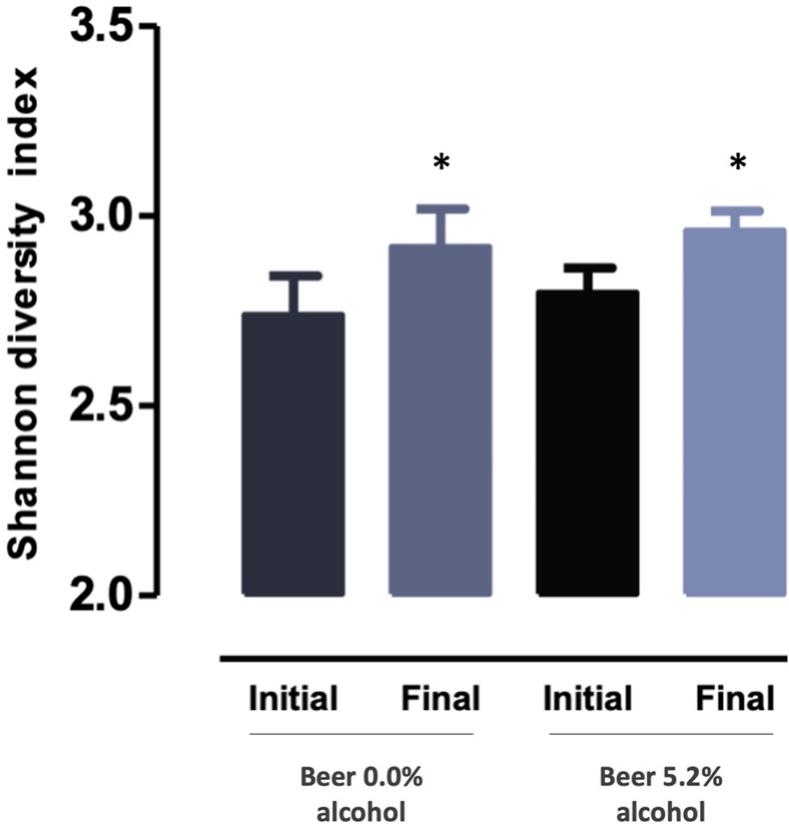
Microbial diversity measured by Shannon’s diversity index in the two intervention groups, at baseline (initial) and 4 weeks after intervention (final). Values are expressed as mean ± standard deviation (*n* = 9–10). *P < 0.05 vs initial.

Neither nonalcoholic nor alcoholic beer induced significant differences in specific gut bacterial phylum and genus (*p* > 0.05). Accordingly, the PCoA plot (Figure 4) suggest similarity between each time point, in both groups. Nevertheless, nonalcoholic and alcoholic beer increased the bacterial diversity as determined by Shannon diversity index, from 2.7 ± 0.3 to 2.9 ± 0.3, *P* = 0.037 and from 2.8 ± 0.2 to 3.0 ± 0.2, *P* = 0.021, respectively (Figure 5).

### Fecal Alkaline Phosphatase (ALP) Activity

**Figure 6:**
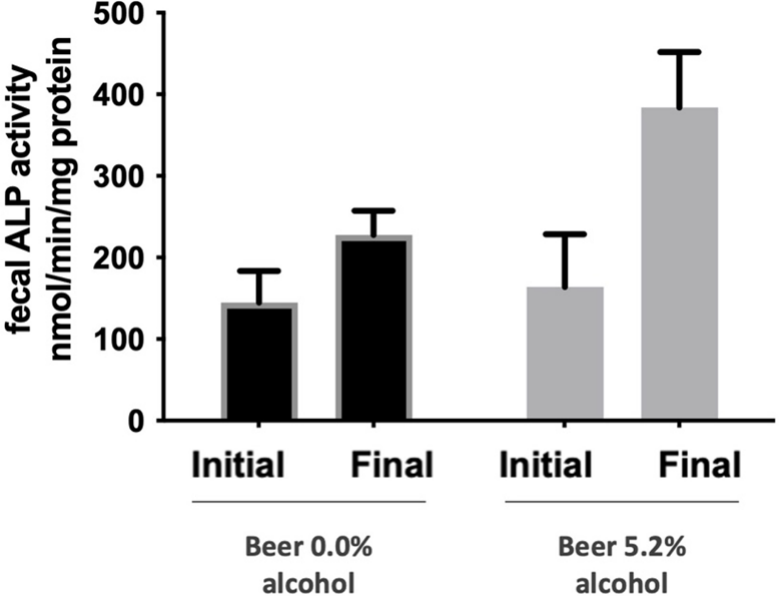
Fecal alkaline phosphatase (ALP) activity. Values are expressed as mean ± standard error of mean (*n* = 9–10).

Nonalcoholic beer and alcoholic beer tended to increase fecal ALP activity from 156 ± 116 to 230 ± 114 nmol/min/mg protein, *P* = 0.051 and from 183 ± 216 to 429 ± 227 nmol/min/mg protein, *P* = 0.051, respectively (Figure 6).

## Discussion

In the present study, we evaluated the effects of drinking 1 bottle (330 mL) of nonalcoholic and alcoholic beer per day, for 4 weeks, on serum cardiometabolic markers and gut microbiota composition in healthy men. Results from this study show that drinking nonalcoholic beer or alcoholic beer increase gut bacterial diversity, without significantly change body weight, body fat mass, and serum cardiometabolic markers.

Decreased bacterial diversity has been associated with diabetes and cardiovascular disease.^16^ In addition, it has been shown by our group that decreased bacterial diversity increased the risk for severe COVID-19, for which obesity and diabetes are important risk factors.^12^ Therefore, promoting changes in the gut microbiota to correct dysbiosis and increase bacterial diversity may mediate the effects of successful dietary interventions in the prevention of these chronic diseases.^14^

Fermented beverages such as red wine have been shown to induce favorable changes in the gut microbiome due to their high polyphenol content.^17^ In fact, red wine is a source of anthocyanins which have been shown to modulate the gut microbiota composition.^11^ However, beer includes a range of polyphenols such as flavonoids and phenolic acids and is the richest dietary source of isoxanthohumol. These phenolic compounds may contribute to the increase in bacterial diversity observed in the gut microbiota of participants that consume either alcoholic or nonalcoholic beer. These results are in accordance with other recent study showing that nonalcoholic beer (0.5% v/v alcohol) consumption for 30 days increases gut microbial alpha-diversity.^18^ During beer production, especially during beer filtration (often conducted to clarify the beer), important compounds can be removed, including polyphenols, yeast and yeast components. Thus, beers with higher amounts of polyphenols and yeast (e.g., nonfiltered beers) may even have a greater impact on the gut microbiome than the Lager beers used in the present study.

Alcohol consumption has been shown to decrease bacterial diversity,^19^ however, in our study the consumption of alcoholic beer increased gut bacterial diversity. Thus, beer polyphenols seem to have surpass the deleterious effect of alcohol on the gut microbiome. In the study of Hernandez-Quiroz et al., the moderate consumption of alcoholic beer did not increase gut bacterial diversity.^18^ However, the crossover design of the study as well as the differences in the baseline microbiome of the population under study (Mexican vs Portuguese population) may contribute to explain the discrepancy in the results obtained.

Our results also showed that serum ALP is decreased after 4 weeks of daily beer intake, independently of beer alcoholic content. A former study investigating the modulation of ALP activity in vascular smooth muscle cells by polyphenols rich beverages, concluded that “Lager” type beer had a strong inhibitory effect on ALP activity.^20^ Results on serum (tissue nonspecific) ALP activity may not have clinical significance since this biomarker is usually used to evaluate liver, bone, or heart damage when ALP activity is elevated. Nevertheless, it would be interesting to further investigate what caused a reduction in serum ALP activity and if it is associated to improved liver, bone or heart function.

When analyzing the activity of ALP in fecal samples, we observed that both nonalcoholic and alcoholic beer tended to increase fecal ALP activity. As recently proposed by our group, ALP activity may have increased due to the increased production of butyrate.^21^ Butyrate is well-known to induce ALP activity and although no specific changes in butyrate-producing bacteria were observed in the present study after the consumption of nonalcoholic and alcoholic beer, since fecal concentrations of short chain fatty were not measured, this hypothesis cannot be ruled out. Increased fecal ALP activity may be indicative of improved intestinal barrier function, since ALP dephosphorylates lipopolysaccharide which contributes to reduce inflammation and intestinal permeability.^22^ In addition, fecal ALP activity has been shown to reduce the risk of type 2 diabetes.^23^ Thus, although in the present study, we did not observe significant changes in serum cardiometabolic biomarkers, these effects of beer on gut microbiota modulation and ALP activity suggest a beneficial effect on health and deserve to be investigated in a population with metabolic disease.

This study has some limitations that should be addressed. Since this is a pilot study, our hypothesis should be proven in the next large-scale parallel group comparison study. In addition, the effects of nonalcoholic beer on gut microbiota diversity should be further evaluated in participants that do not usually drink alcohol, in comparison with low polyphenol-content carbonated drink.

The activity of alcohol dehydrogenase (ADH) and aldehyde dehydrogenase (ALDH) was not evaluated in the group of participants drinking alcoholic beer. Nevertheless, the main aim of the present study was to evaluate the effects of beer with and without ethanol on the gut microbiota composition and diversity. Ethanol may interfere with the absorption of polyphenols and, consequently, with the amount of polyphenols that reach the gut and interact with the gut microbiota. Thus, we considered that even if there are differences in alcohol metabolism between our study participants, after absorption, it does not affect our primary outcome and main results.

In conclusion, our data obtained from a randomized, two arm parallel-group trial show that both nonalcoholic and alcoholic beer increase gut microbiota diversity, after 4 weeks of daily consumption, without causing any significant change in weight, BMI, and cardiometabolic markers, making these beverages an interesting approach to increase microbiota diversity.

## Supporting Information Available

Table S1, nutritional composition of the beers under study (PDF)
