# Supplementary material for: Impact of Beer and Nonalcoholic Beer Consumption on the Gut Microbiota: A Randomized, Double-Blind, Controlled Trial
Source: J Agric Food Chem. 2022 Jun 15;70(41):13062–70. doi: 10.1021/acs.jafc.2c00587 (PMC9776556; doi:10.1021/acs.jafc.2c00587)
Supplement: Supplementary file 1 — jf2c00587_si_001.pdf [file jf2c00587_si_001.pdf]

**Table S1 -** Nutritional composition of beers under study.

| Units/100 ml | Method | Beer 0.0% alcohol | Beer 5.2% alcohol |
|---|---|---|---|
| Energy (kcal) [1)] | Reg(UE)1169/11 | 26 | 39.8 |
| Total Fat (g) [1)] | PAFQ 069B.3 | <0.1 (LOQ) | <0.1 (LOQ) |
| Total Carbohydrates (g) [1)] | Reg(UE)1169/11 | 5.9 | 2.8 |
| Sugars (g) [1)] | AOAC 920.51 | 1.6 | 0.6 |
| Protein (g) [1)] | AOAC 920.53 | 0.55 | 0.37 |
| Fiber (g) [1)] | PAFQ 230.2 | <0.3 (LOQ) | <0.3 (LOQ) |
| Vitamin B6 (mg) [1)] | PNTA 0056 | 0.08 | 0.06 |
| Vitamin B9 (µg) [1)] | PNTA 0138 | 3.0 | 1.1 |
| Vitamin B12 (µg) [1)] | PNTA 0137 | <0.1(LOQ) | <0.1(LOQ) |
| Calcium (mg) | [2)] | 5.2 ± 0.14 | 3.4 ± 0.07 |
| Sodium (mg) | [2)] | 3.6 ± 0.00 | 3.0 ± 0.01 |
| Potassium (mg) | [2)] | 62.9 ± 0.38 | 43.3 ± 0.05 |
| Phosphorus (mg) | [2)] | 43.3 ± 0.94 | 31.1 ± 0.21 |
| Magnesium (mg) | [2)] | 10.7 ± 0.09 | 7.6 ± 0.00 |
| Xanthohumol (µg/L) | | < 0.8 (LOQ) | < 0.8 (LOQ) |
| Isoxanthohumol (µg/L) | | 50.25 | 59 |
| Total Phenolics (mM gallic acid equivalents) | | 2132 | 1566 |

LOQ - Limit of quantification.

[1)] Taken from the nutritional label of the beers under study

[2)] Minerals were determined according to the following methodology:

About 2 g of beer sample were mixed in a clean vessel with 2 mL hydrogen peroxide (30%, VWR BDH chemicals, France) and 6 mL nitric acid (67-69%, VWR BDH Prolabo, Canada). Heated digestion was performed by microwave (Milestone model Ethos one, Sorisole, Italy) for about 47 min under controlled temperature and predetermined power (800W). The first 15 minutes, temperature was raised to a peak of 180ºC, and maintained between 120ºC to 180ºC during 12 min, and then cooled down. After the digestion, water was added to perform a volume of 50 mL. Flame atomic absorption spectrophotometry (AAS) (ICE™ 3000, Thermo Scientific, Unicam, Lisboa, Portugal) was used to quantify Sodium (589 nm),

Potassium (766,5 nm) and Calcium (422,7 nm). Inductively Coupled Plasma - Optical Emission Spectroscopy (ICP-OES) (Activa M, Horiba Jobin Yvon, France), with a radial plasma configuration and a concentric quartz nebulizer was used to quantify phosphorous (213,618 nm) and magnesium (279,553 nm). Calibration was performed using a Sodium and Calcium AAS standard of 1g/L in 2% HNO3 (AVS Titrinorm, VWR BDH Prolabo), and all other minerals by a multi-elemental standard solution in 5% HNO3 (PlasmaCAL, SCP Science, Canada) was used to prepare calibration standards. All samples were digested and quantified in duplicates and results were given in mg/L. Additionally, deviations were checked using fortified samples and a reference sample. All tubes used were previously immersed in dilute HNO3 solution overnight, then cleaned with ultrapure water, and oven-dried.

Xanthohumol and isoxanthohumol were measured by HPLC-DAD after SPE extraction, in Faculty of Pharmacy of University of Porto and total phenolics were determined by Folin-Ciocalteu method
